# Dietary considerations in the evaluation and management of nocturia

**DOI:** 10.12688/f1000research.21466.1

**Published:** 2020-03-05

**Authors:** Upeksha S Alwis, Thomas F Monaghan, Rebecca Haddad, Jeffrey P Weiss, Saskia Roggeman, Erik Van Laecke, Johan Vande Walle, Alan J Wein, Karel Everaert

**Affiliations:** 1 Department of Human Structure and Repair, Ghent University Hospital, Ghent, Belgium; 2 Department of Urology, State University of New York Downstate Health Sciences University, Brooklyn, New York, USA; 3 Sorbonne Université, UPMC Univ Paris 06, AP-HP, GRC 01, Groupe de Recherche Clinique en Neuro-Urologie (GREEN), Service de Rééducation Neurologique, AP-HP, Paris, France; 4 Department of Urology, Ghent University Hospital, Ghent, Belgium; 5 Department of Pediatric Nephrology, Ghent University Hospital, Ghent, Belgium; 6 Department of Urology, Perelman School of Medicine at the University of Pennsylvania, Philadelphia, PA, USA

**Keywords:** Nocturia, Food, Diet, Nutrients

## Abstract

**Aim: **This narrative review investigates the effect of dietary intake on nocturnal voiding severity. The primary aims of this review are to provide a framework for future research and ultimately contribute to more comprehensive, lifestyle-centered guidelines for the management of nocturia.

**Methods: **A literature search was conducted in Web of Science, PubMed, and Google Scholar databases using the keywords “nocturia”, “diuresis”, “natriuresis”, “food”, “diet”, and “nutrients”.

**Results:** High fruit and vegetable consumption was negatively associated with nocturia. High intake of tea and dietary sodium showed a positive association with nocturia. Several foods have also been directly linked to changes in diuresis rate, glycemic control, and endogenous serum melatonin concentration, offering potential mechanisms for this observed effect. Overall quality of the evidence was low.

**Conclusion** **:** At present, there is limited evidence to suggest that certain foods, electrolytes, and specific compounds may contribute to the pathogenesis of nocturia. A greater understanding of the impact of food and nutrients on body fluid metabolism is needed to further refine the evaluation and treatment of nocturia.

## Introduction

Nocturia, defined as the act of waking to void during the hours of intended sleep, is among the most common and bothersome lower urinary tract symptoms (LUTSs) ^1^. Although the prevalence of nocturia increases with age ^2^, nocturia is a common complaint among both men and women of all ages and backgrounds ^3^. Nocturia is associated with an increased mortality rate and morbidity, owing largely to its direct negative impact on sleep architecture and daytime function and its role as an independent risk factor for falls and hip fractures in the elderly ^2^.

Reducing fluid intake and caffeine and alcohol intake and improving sleep hygiene are involved in lifestyle modification in nocturia management ^2, 4^. In cases of nocturia refractory to conservative measures, the management of nocturia centers on a broad range of pharmacologic therapies, including antimuscarinics in the setting of concomitant overactive bladder (OAB), α-blockers and 5α-reductase inhibitors for men with benign prostatic hyperplasia (BPH), medication for improving sleep (for example, sedatives and melatonin), and antidiuretic therapy ^2, 4^.

However, in the progression from initial individualized behavioral modification and lifestyle interventions to pharmacologic treatments, the role of food and other nutrients is often overlooked. Urine is produced in order to regulate the body fluid homeostasis, electrolytes, the acid–base balance and to remove toxins and by-products ^5^. Food and beverages which are the main dietary source of fluids, electrolytes (for example, sodium, potassium, or calcium), and other osmotically active substances (for example, urea and glucose) can therefore promote diuresis, contributing to nocturnal or 24-hour polyuria or both. Accordingly, the objective of this narrative review is to evaluate available data on the effect of dietary intake on the prevention and treatment of nocturia.

## Methodology

This narrative review was based on a literature search in Web of Science, Embase, Medline, and Google Scholar databases with the keywords “nocturia”, “LUTS”, “diuresis”, “natriuresis”, “food”, “diet”, and “nutrients”. The search was limited to articles published in the English language. No time limits were applied. Randomized controlled trials (RCTs), prospective observational studies, retrospective series, case reports, editorials, research letters, review articles, meeting abstracts, textbooks, and book chapters were included for this narrative review.

## Results

### Diet and nocturia

**Figure 1.**
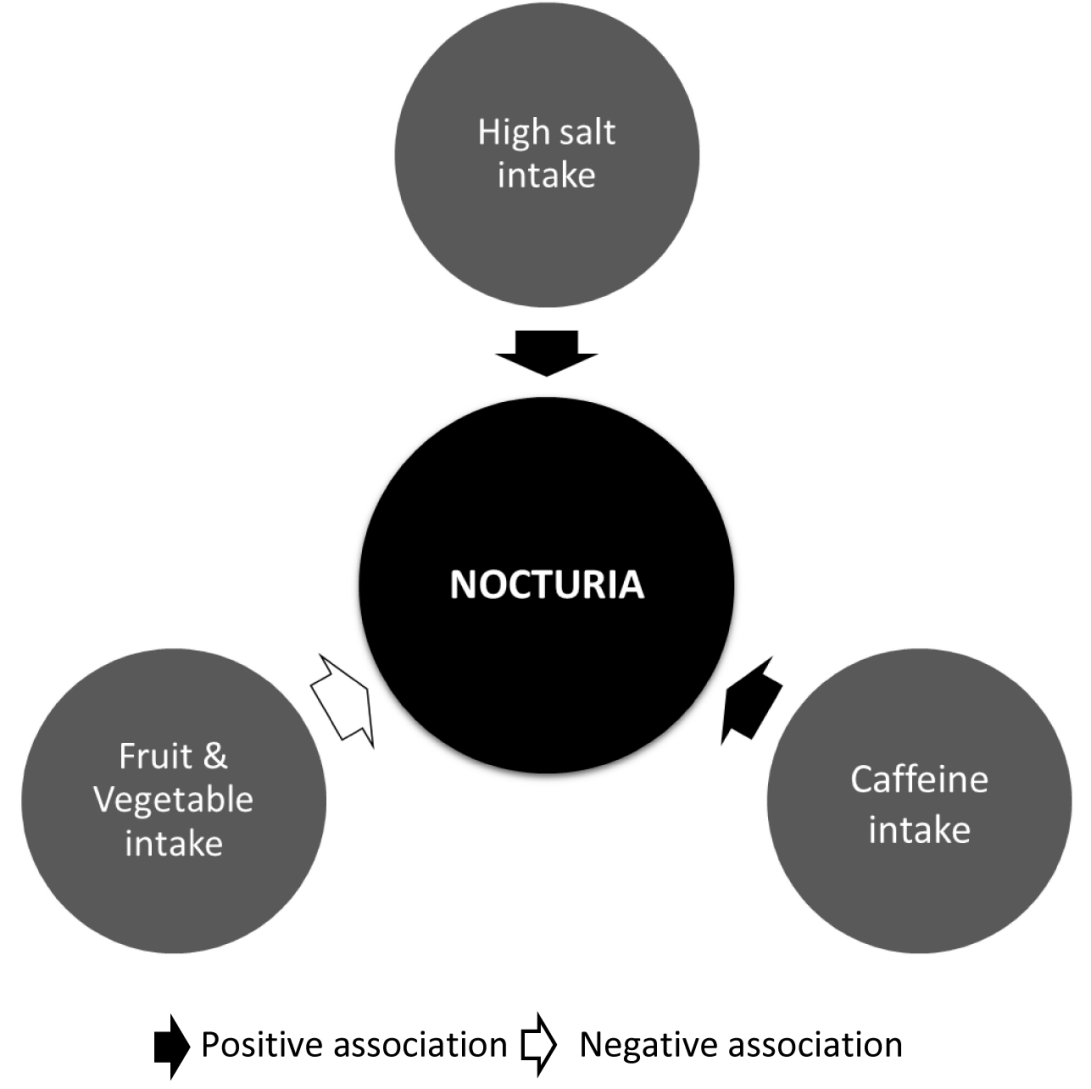
Effect of different types of food and beverages on nocturia.

A summary of the effect of different types of food and beverages on nocturia is provided in Figure 1.

***Fluid intake.*** Although food moisture constitutes a sizable minority of the total dietary fluid load ^6^, the cumulative volume of all drinks consumed throughout the day constitutes a majority of total body water, and fluid intake volume is highly correlated with urine volume ^7^. Moreover, a decrease in dietary fluid intake has been shown to significantly improve nocturia severity in well-controlled randomized prospective trials ^8^. Consistently, individualized behavioral modification counseling has been shown to be an effective intervention in the treatment of clinically significant nocturia ^9, 10^, and expert consensus from the International Continence Society has indeed endorsed fluid management as being central to the initial evaluation and management of nocturia ^11^.

***Sodium intake.*** High dietary sodium intake leads to increased thirst and subsequent fluid intake. Thus, dietary sodium restriction is an increasingly popular lifestyle recommendation for improving nocturia severity ^12^.

This effect of dietary sodium restriction on nocturnal voiding frequency has been shown in a non-RCT of elderly Japanese patients (n = 321) with at least one nocturnal voiding episode and high dietary salt intake (≥8 g/day for men and ≥7 g/day for women) ^13^ at baseline. A significant reduction in nocturia (2.3 ± 0.9 to 1.4 ± 1.0 voids) was observed in subjects who successfully reduced their mean salt intake from 10.7 to 8.0 g/day. Conversely, subjects whose mean salt intake increased from 9.6 to 11 g/day incurred a significant increase in nocturnal voiding severity (2.3 ± 1.1 to 2.7 ± 1.1 voids) ^13^.

High dietary sodium intake in relation to nocturia has also been investigated in observational protocols. A cross-sectional clinical study evaluated the relationship between the daily salt intake (high salt [11.4 g/day] versus low salt [7.3 g/day]) and nocturia in 728 patients ^14^. Both daytime and nighttime voiding frequencies were significantly higher among the group with high sodium intake (8.4 ± 2.4 and 2.2 ± 1.3, respectively) compared with the low-salt group (6.9 ± 2.5 and 1.4 ± 1.3, respectively). Daytime and nighttime urine volumes among the high-salt group (1811.7 ± 477.7 and 517.7 ± 241.1 mL, respectively) were significantly higher compared with those among the low-salt group (1590 ± 502.3 and 153.7 ± 146.8 mL, respectively) ^14^.

Dietary salt intake is highly dependent on cultural, socioeconomic, and geographical factors and intra-individual dietary habits. However, the predominant source of dietary sodium tends to be attributable to processed foods (70–75%) ^15^, whereas salt added for flavoring or during cooking (10–15%), coupled with that naturally present in unprocessed foods (10–15%) ^15^, typically constitutes but a minority of total dietary sodium intake. Reduction in dietary salt intake might be beneficial for nocturia patients with high salt intake—particularly in those who respond poorly to urologic medications ^13^—which involves careful consideration of all potential sources of excess dietary sodium.

***Fruits and vegetables.*** Fruits and vegetables are rich sources of dietary nutrients such as vitamins, minerals, and fibers and many bioactive phytochemicals such as antioxidants and polyphenols ^16^.

A longitudinal cohort study evaluated the association between consumption of fruits and vegetables and development of LUTS based on a validated Chinese version of the International Prostate Symptom Score (IPSS) ^16^ for LUTS severity. Specifically, IPSS was assessed in 1564 elderly Chinese men at baseline and at 4-year follow-up encounters ^16^. High fruit and vegetable intake (>350 g/1000 kcal per day) significantly reduced the “storage LUTS” (urinary frequency, urgency, and nocturia) (IPSS: −0.448 ± 0.230) compared with the moderate intake of fruits and vegetables (250–350 g/1000 kcal per day). High intake of dark leafy vegetables (>50 g/1000 kcal per day) likewise significantly reduced storage symptoms (IPSS: 0.347 ± 0.183) compared with moderate intake (25–50 g/1000 kcal per day) ^16^. Furthermore, compared with moderate intake, high intake of dark leafy vegetables significantly reduced the risk of incident storage symptoms (odds ratio (OR) 0.666, 95% confidence interval (CI) 0.488–0.907). No association was observed between intake of soy foods, cruciferous vegetables, tomatoes, citrus fruits, and storage symptoms ^16^.

Similarly, Furukawa *et al*. identified a negative association between vegetable intake (frequency) and nocturia in Japanese patients with type 2 diabetes (mean age of 61.7 years) ^17^. In that prospective cohort study of 785 patients, increased vegetable intake habit was protective for clinically significant nocturia (at least two voids per night; OR 0.67, 95% CI 0.48–0.94) and for severe nocturia (at least three voids per night; OR 0.46, 95% CI 0.30–0.71) ^17^. However, this study could not assess the dose–response relationship between vegetable intake and nocturia.

***Caffeine intake.*** Caffeine belongs to the methylxanthine family of compounds and is present in a wide variety of foods and beverages ^18^. Caffeine is 100% bioavailable and excreted through urine ^18^. Caffeine is known to increase lower urinary tract smooth muscle contractility and stimulation of the central nervous system, and both mechanisms contribute to increased diuresis ^19^. A cohort study on the effect of caffeinated beverages on urinary incontinence among Swedish female twins (n = 14,031) reported that high tea consumption (>3 cups per day) was associated with an increased risk of nocturia (defined as at least two voids per night; OR 1.18, 95% CI 1.01–1.38), but no association was observed between coffee consumption and nocturia ^19^.

### Dietary effect on 24-hour polyuria

***Glycemic control.*** Many studies have reported on traditional foods, spices, and medicinal plants used by alternative medical customs and belief systems in the treatment of LUTS ^20– 27^. A non-RCT studied the effect of an Ayurvedic diet (traditional Indian medicine) on 30 patients with diabetes ^20^. The diet was prepared on the basis of Ayurvedic diet, which included cereals (barley), legumes, vegetables, fruits, nuts and seeds, oils, and animal protein (eggs and milk), along with herbs, spices, herbal supplements, teas, and medicated milk (milk prepared with herbs). Following treatment, significant improvements were observed in clinical symptoms scores for polyuria (1.3 ± 1.022 to 1.06 ± 1.04), polydipsia (1.1 ± 0.959 to 0.861 ± 0.86), and polyphagia (1.2 ± 0.886 to 1.06 ± 0.944) ^20^.

***Diuretic action of food.*** There is some expert opinion regarding certain foods/herbs in relation to urine production. In medieval Persian and Arabic medical classics, grass of Parnassus, blue leek, chickpea, wild rocket, cabbage, onion, cinnamon, Celtic spikenard, celery, and wild iris are referenced as highly effective diuretics ^24^. In Ayurveda classics, a group of 10 herbs named “mutravirechaniya mahakashaya” (great extractives of diuretics) promised to effectively cure several common urinary disorders, including increased urinary frequency, urinary tract infections, and renal calculi ^28^. Furthermore, plants containing cardiac glycosides or methylxanthines (coffee and tea) or plants with angiotensin-converting enzyme (ACE)-inhibiting activity (garlic, tea, and olive) have been reported for their potential diuretic activity ^22, 23, 26^. However, very little knowledge is available on the potential bidirectional relationship between foods and diuresis.

### Dietary effect on sleep

Melatonin is one of the key regulatory hormones of circadian rhythms ^29^. A dysregulation in the release of this hormone is involved in sleep disorders ^30^. Low serum melatonin levels have been observed in patients with nocturia compared with patients without nocturia ^31^. In a cross-sectional cohort study of 861 elderly men, those with a higher urinary concentration of 6-sulfatoxymelatonin (6-SMT) (the main metabolite of melatonin in the urine) incurred significantly lower odds for nocturia (adjusted OR 0.73, 95% CI 0.56–0.96) ^29^.

Exogenous melatonin supplementation has been used to improve both sleep quality and nocturia ^2, 29, 31^. A randomized clinical trial of 42 elderly patients with nocturia compared melatonin (2 mg/day) with a sedative, rilmazafone hydrochloride (2 mg/day) ^31^. The mean number of nocturnal voiding episodes significantly decreased in both the melatonin- and rilmazafone-treated groups (3.4 to 2.6 and 3.5 to 2.5 nocturnal voids, respectively). In a crossover RCT, 20 elderly men with bladder outflow obstruction (BOO) and nocturia (mean of 3.1 nocturia episodes per night) were treated with 2 mg melatonin ^30^. Following treatment, nocturia decreased in both the intervention and placebo groups (0.32 and 0.05 nocturnal voids, respectively). The rate of nocturia treatment response, defined as a reduction from baseline of at least 0.5 episodes per night, was significantly higher in the intervention group ^30^.

Importantly, foods rich in nutrients are important in the synthesis of endogenous melatonin. Indeed, some of them act as precursors, cofactors, and activators in the production of this hormone, including tryptophan, B vitamins, magnesium, zinc, and polyunsaturated fatty acids. Furthermore, foods such as rice, barley, tomatoes, cranberry, strawberry, walnuts, olive oil, unprocessed cow milk, eggs, and fish are rich dietary sources of bioavailable melatonin ^32, 33^.

An increased concentration of melatonin in the blood and urine after ingestion of dietary melatonin has been reported in both animal and human studies ^32^. A crossover study investigated the impact of dietary melatonin on serum melatonin concentration (SMC) in 12 healthy male subjects ^34^. Participants consumed orange or pineapple juice (from 1 kg of fruits) or two whole bananas. Peak SMC levels were observed 120 minutes following fruit consumption and returned to baseline values 180 minutes after ingestion. SMC values significantly increased from baseline for the pineapple juice (48 versus 146 pg/mL), orange juice (40 versus 151 pg/mL), and banana (32 versus 140 pg/mL) ^34^.

Specific dietary habits, foods, and compounds have also been associated with changes in endogenous SMCs. Energy restriction (<300 kcal per day) has been shown to reduce nocturnal melatonin concentration by 20%. Caffeine has been reported to possess both stimulatory and inhibitory effects on melatonin level ^32^. Alcohol consumption has been shown to reduce nocturnal SMC ^32^.

## Discussion

Lifestyle interventions are an important first-line treatment for voiding dysfunction. Knowledge of therapeutic action of foods, spices, herbs, and minerals is commonly used by many alternative and complementary medicine systems ^24, 35^. Moreover, the International Consultation on Incontinence has recognized the need for increased research on the conservative management of voiding dysfunction ^2^. This study evaluated the dietary impact on nocturia as synthesized from currently available literature. Overall, there is evidence to suggest that certain foods, electrolytes, and specific compounds may contribute to the pathogenesis of nocturia, but there remains poor overall evidence with regard to specific conclusions and largely insufficient evidence to establish causality.

Nevertheless, this synthesis of current literature overviews several promising avenues for future investigation, particularly with respect to dietary sodium and fruits and vegetables. High dietary sodium intake increased nocturia in a non-RCT ^13^, and this observation was further supported in an observational study ^14^. High dietary intake of fruits and vegetables showed a negative association with nocturia, whereas high intake of tea showed a positive association with nocturia in observational studies.

Several potential mechanisms may contribute to the observed associations identified in this review. Fruits and vegetables are rich in antioxidant phytochemicals ^17^ and thus may have a beneficial effect on nocturia, particularly in the setting of OAB, bladder outlet obstruction secondary to BPH, and other urinary storage symptoms, which are important contributors to nocturia that may be mediated by inflammation ^36, 37^. Vegetable intake has also been reported to reduce post-prandial hyperglycemia, which is likewise associated with decreased oxidative stress and inflammation ^17^. Thus, intake of vegetables might protect the prostate and bladder by preventing inflammation and oxidative damage ^17^. Notably, intake of vegetables, fruits, vegetable fats, citrus juice, pumpkin seeds, bread, chicken, dietary isoflavone, and beer and intake levels of vitamin D, protein, and potassium demonstrated significant negative associations with OAB and BPH ^16, 17, 38^.

Moreover, the relationship between foods and diuresis may be indirectly mediated by melatonin. Melatonin is a derivative of the essential amino acid tryptophan ^33^. It is a neurotransmitter hormone that is critical for sleep maintenance and the regulation of circadian rhythm (day–night/sleep–wake cycle) ^29, 31, 32^. Nighttime urination frequency follows this biological rhythm, as characterized by decreased urine production and increased bladder storage from the daytime to nighttime period ^29^. Intake of cereals, vegetables, fruits, caffeine, and certain vitamins and minerals may modify melatonin production in the body ^32, 34^. Therefore, insufficiency in dietary melatonin-rich foods or dietary nutrients may compromise melatonin production in the body—especially in older adults since the production of melatonin decreases with age ^32^. Future research is needed to explore the potentially significant benefits of dietary melatonin in nocturia patients with low serum melatonin.

Beyond this conventional wisdom that certain foods have diuretic and therapeutic properties, a wide range of phytochemicals such as phenolic compounds (flavonoids and tannins), alkaloids, coumarins, saponins, and glycosides have been reported for their potential diuretic activity through ACE inhibition or Na ^+^/K ^+^/ATPase-inhibiting activity ^23^. Moreover, Liu *et al*. suggested that the observed beneficial association between fruit and vegetable intake and LUTS was not attributable to single micronutrients but to a collective effect and interactions of different nutrients available in the food ^16^.

This narrative review highlights substantial areas for growth in the study of the diuretic properties of foods and beverages. Namely, the relationship between diet and nocturia must be interpreted in view of concomitant LUTS and systemic comorbidities such as obstructive sleep apnea which may influence the clinical phenotype of a patient’s nocturnal voiding dysfunction ^39, 40^. Moreover, substantial dietary changes have been described to occur across the developmental period and throughout adulthood ^41^ while the aging process itself may influence the pathogenesis of nocturia among patients with clinically relevant symptoms ^42^.

## Conclusions

At present, there is evidence to suggest that certain foods, electrolytes, and specific compounds may contribute to the pathogenesis of nocturia, but there remains poor overall evidence with regard to specific conclusions and largely insufficient evidence to establish causality. A greater understanding of the impact of food and nutrients on body fluid metabolism is important for improving the evaluation and treatment of nocturia. This area of study may also have direct implications with respect to other nocturnal voiding disorders such as enuresis, in which excess nocturnal urine volume is likewise a prominent underlying mechanism.
